# Rewiring the Vehicle: *Trypanosoma cruzi* Parasites Alter the Antennae of Their Triatomine Hosts

**DOI:** 10.1002/ece3.71164

**Published:** 2025-03-23

**Authors:** Jose D. Rivera‐Duarte, Irving Jesús May‐Concha, Reyna Vargas‐Abasolo, Mayab X. Martínez‐Castaneira, Manuel Edday Farfán‐Beltrán, Berenit Mendoza‐Garfias, Any Laura Flores‐Villegas, Alex Córdoba‐Aguilar

**Affiliations:** ^1^ Departamento de Ecología Evolutiva Instituto de Ecología, Universidad Nacional Autónoma de México, Circuito Exterior, Ciudad Universitaria Ciudad de México Mexico; ^2^ Laboratorio de Hidrobiología, Departamento de Ecología y Recursos Naturales, Escuela de Biología, Facultad de Ciencias Universidad Nacional Autónoma de Honduras, Ciudad Universitaria Tegucigalpa Honduras; ^3^ SECIHTI, Centro de Investigaciones Regionales Dr. Hideyo Noguchi Universidad Autónoma de Yucatán Mérida Mexico; ^4^ LMF 1‐LANABIO, Instituto de Biología Universidad Nacional Autónoma de México, Circuito Exterior, Ciudad Universitaria Ciudad de México Mexico; ^5^ Departamento de Microbiología y Parasitología, Facultad de Medicina Universidad Nacional Autónoma de México, Ciudad Universitaria Ciudad de México Mexico

**Keywords:** antennal phenotype, chagas disease, parasite‐host relationship, sensory mechanisms, Triatominae

## Abstract

This study investigates the antennal phenotype of the kissing bug *Triatoma pallidipennis* (Stål), a primary vector of Chagas disease, by comparing *Trypanosoma cruzi*‐infected and noninfected individuals. We examined the antennae of infected and noninfected N5 nymphs, as well as adult females and males, focusing on four types of sensilla (bristles, basiconic, thin‐walled trichoid, and thick‐walled trichoid) across three antenna segments (pedicel, proximal flagellum, and distal flagellum). We found differences in sensilla abundance across the antennal segments, with the proximal flagellum showing the highest abundance, followed by the distal flagellum, and the pedicel having the least. Infection demonstrated that males had more chemosensilla than females. We observed a trend in the infected males and nymphs with an increased variation in sensilla types. These antennal modifications are related to previous results in this species whereby infected bugs were found to be more active and capable of finding a human odor compared to noninfected animals. Thus, infection‐related changes in antennal phenotype may underlie *T. pallidipennis* ' sensory capabilities, which may indirectly facilitate the spread of the parasite.

## Graphical abstract

Parasite *Trypanosoma cruzi* infects triatomine bugs. Infection induces antennal sensillum change in distribution and number in bugs. Antennal sensillum changes can make the bugs' search for food more efficient.

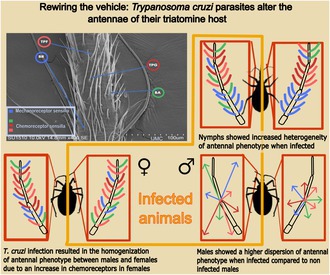

## 1 Introduction

The relationship between a parasite and its host is usually understood as an evolutionary arms race involving continuous adaptations by both organisms (Buckingham and Ashby 2022). One emerging feature of this race is that parasites may change the ecology, physiology, morphology, and/or behavior of their hosts to increase parasite fitness (Thomas et al. 2010). For example, parasitic plants can induce root hypertrophy in their hosts, which helps them to colonize the host more efficiently (Spallek et al. 2017). A fascinating area that has been recently uncovered pertains to how parasite‐driven effects may shape hosts' communication by altering their sensory system (Reichert et al. 2023).

One instance of a parasite effect that has come to light recently is the one that causes Chagas disease. A key player here is the Chagas‐causing parasite *Trypanosoma cruzi*, which requires two host types to complete its life‐cycle complex: Triatomine “kissing bugs” (Hemiptera: Reduviidae) and mammals. Kissing bugs are mainly blood feeders, relying on vertebrate hosts, including humans, for food (they may also feed on hemolymph, as demonstrated by Otálora‐Luna et al. 2021). Infection with *T. cruzi* enhances the ability of bug hosts to detect mammal odors (Ramírez‐González et al. 2019), search more actively for food (Botto‐Mahan 2009; Chacón et al. 2022), and increase biting, thus allowing the parasite to come into contact with the mammal hosts more quickly (Chacón et al. 2022; Pereyra et al. 2020). Therefore, the parasite effect is directed at enhancing the ability of kissing bugs to find and make use of hosts. Note, however, that other studies have found no significant effects of *T. cruzi* infection on feeding patterns (Takano‐Lee and Edman 2002), gut microbiota (Omondi et al. 2024), development and reproduction (Botto‐Mahan et al. 2017; Lima et al. 1992; Schaub 1988), or toxicological responses to chemical agents (Lobbia et al. 2023). Furthermore, the effect of the parasite on its triatomine host may be limited or highly dependent on factors such as species, age, sex, and environmental conditions (May‐Concha et al. 2022; Schaub 2021).

The insect sensory system is the first step for host detection. It consists of a plethora of highly diverse small organs called sensilla. In triatomines, two types of sensilla have been identified: Mechanoreceptors, which are sensitive to mechanical stimuli such as vibration and changing air currents, and chemoreceptors, which are responsible for detecting chemical stimuli related to host sources and the location of sexual partners (Catala 1997; May‐Concha et al. 2022). Interestingly, the distribution of sensillum types on the antennae of triatomines, including their density and spatial arrangement (i.e., antennal phenotype), is linked to environmental conditions (Dujardin et al. 2009; May‐Concha et al. 2016). For instance, urban populations of *Triatoma dimidiata* tend to have fewer antennal sensilla than sylvatic populations, presumably because less complex habitats have been selected for less complex neuronal arrangements (Montes de Oca‐Aguilar et al. 2022). Previous research has identified significant differences in the antennal phenotype of *Triatoma dimidiata* individuals based on their infection status (see May‐Concha et al. 2022). Although the authors were unable to establish a functional relationship (e.g., that more sensilla are needed for complex habitats), they reported a strong association between natural infection and variations in antennal phenotype. Therefore, the extent to which the antennal phenotype is sensitive to *T. cruzi* infection is a likely yet unexplored possibility. Using an experimental approach where we controlled *T. cruzi* infection, we investigated the resulting antennal phenotype of *Triatoma pallidipennis* (Stål) (Hemiptera: Reduviidae) from two developmental stages (N5 nymphs and adults) of both sexes. We hypothesized that there must be morphological differences between infected and noninfected bugs. If found, such differences would be candidates to explain the differential host searching and feeding ability between both groups that have been previously shown in the same study species (Ramírez‐González et al. 2019).

## 2 Materials and Methods

### 2.1 Study Subjects

**FIGURE 1:**
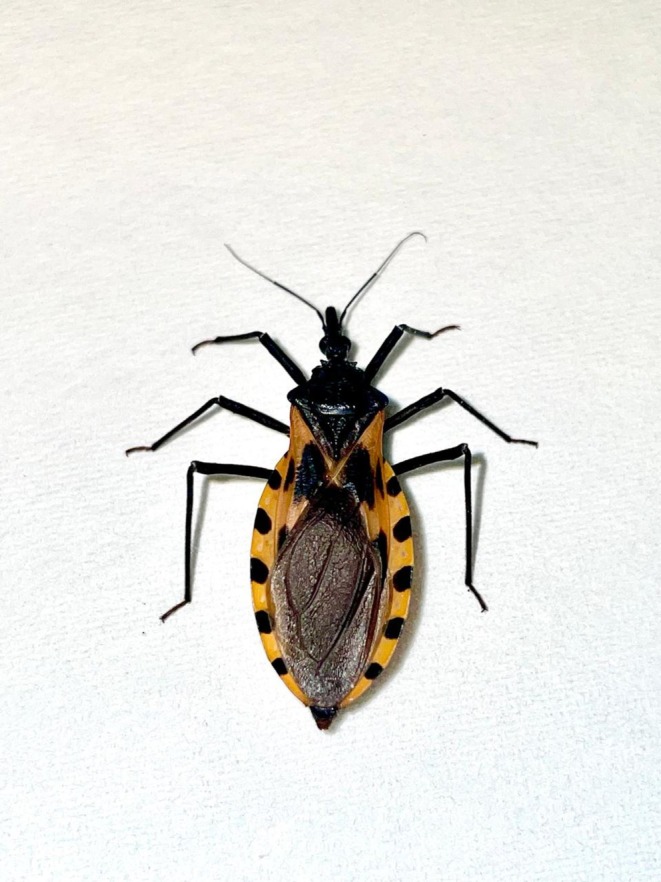
Male adult *Triatoma pallidipennis* , a Mexican chagasic bug (photo taken by A. Córdoba‐Aguilar).

The kissing bug *Triatoma pallidipennis* (Stål) is one of the main vectors of Chagas disease in Mexico (CENAPRECE 2022; Martínez‐Ibarra et al. 2012). *Triatoma pallidipennis* (Figure 1) is distributed across nine states, crossing the western, northwestern, central, and southern regions of Mexico (Meraz‐Medina et al. 2024). This species is commonly found in forest habitats, but its opportunistic nature allows it to infest periurban and urban environments, thriving in disturbed areas with a *T. cruzi* prevalence ranging from 11.3% to 17.7% (Meraz‐Medina et al. 2024).

### 2.2 Insect Rearing

We used *T*. *pallidipennis* specimens from Oaxtepec, Morelos State, Mexico (F15 insect generation). The insects were maintained in controlled temperature, humidity, and light/dark conditions (28°C, 60% RH, and 12:12 h) at the National Institute of Public Health (INSP) insectary and were fed weekly with CD‐1 mice weighing 25–30 g.

### 2.3 Infection of Triatomines With *Trypanosoma cruzi*

Fourth instar kissing bugs were infected with a Morelos isolate (ITRI/MX/12/MOR) of *T. cruzi* , obtained from a *T. pallidipennis* specimen captured in 2012 in Cuernavaca, Morelos. This isolate, characterized as DT1 (sensu Favila‐Ruiz et al. 2018), has been maintained through cyclic passages in CD1 mice. Prior to infection, nymphs underwent a 15‐day fasting period after molting and then were fed on CD‐1 mice weighing 20–25 g, previously inoculated with a concentration of 20,000 parasites/mL. While feeding the insects, we ensured that the mice were in the exponential growth phase of *T. cruzi* by examining a 10 μL sample in a Neubauer chamber. Parasite quantification was performed using a 1:10 dilution in the Neubauer chamber. The triatomines of the control group fed on noninfected mice under identical conditions. For feeding, a group of five insects was fed on a mouse for 20 min. The infection was confirmed 20 days after the insects were fed via extracting and observing the rectal content of the kissing bugs under a light microscope at 40× magnification. The presence of *T. cruzi* showed a successful infection. Subsequently, these insects were monitored throughout their life cycle until their adult phase (Favila‐Ruiz et al. 2018).

### 2.4 Antennal Preparation and SEM Images

We examined 59 antennae of infected and noninfected N5 nymphs, adult females, and males of *T. pallidipennis* (Table 1). Based on the protocol for antennal preparation of May‐Concha et al. (2022), the right antenna was removed from the scape of each specimen (nymph or adult) with fine forceps and scissors. Each antenna was manually cleaned and fixed in a 0.4% glutaraldehyde solution within a phosphate buffer (pH 7.4) for 24 h. Following fixation, the samples were washed multiple times with the buffer and dehydrated using a series of ascending ethanol concentrations (10%, 30%, 50%, 70%, 96%, and 100%). Once dehydrated, the antennas were dried at the critical point using carbon dioxide and mounted on metal stubs using carbon adhesive gold‐coated tabs. Subsequently, they were examined with a Hitachi Stereoscan SU1510 Scanning Electron Microscope (SEM) at 10 kV (Hitachi, Japan) at a magnification of ×400. The number and type of sensilla on the ventral side of the three distal antennal segments (pedicel, proximal flagellum, and distal flagellum) were manually counted. We followed the nomenclature proposed by Catalá and Schofield (1994) to identify the sensilla type, which included bristles, basiconic sensilla, thick‐walled trichoids, and thin‐walled trichoids.

**TABLE 1:** Number of *Triatoma pallidipennis* specimens used in this study, according to sex and infection status.

	Infected	Noninfected	Overall
Female	9	10	19
Male	10	10	20
N5‐Nymph	10	10	20

### 2.5 Data Analysis

We calculated the abundance and average number of sensilla types (bristles, basiconic, thick‐walled trichoids, thin‐walled trichoids) for each antennal segment: pedicel, flagellum 1, and flagellum 2. Then, we used a permutational analysis of variance (PERMANOVA) to compare the composition of antennal sensilla, considering the age, infection status of the insects, their sex, and the interaction between these last two factors. Additionally, we evaluated the homogeneity of multivariate dispersion among the sensilla groups with a permutational analysis of dispersion (PERMADISP), considering both the infection status and sex of the insects. Both analyses were based on Hellinger distance matrices, ideal for abundance data (Legendre and Gallagher 2001), and 999 permutations. We accounted for the sex factor only in adults due to the challenges associated with accurately identifying the sex of triatomines during their nymphal stage.

For univariate analysis, we first calculated the Hill series ^0^ *D*, ^1^ *D*, and ^2^ *D* (sensu Jost 2006) to determine the sensillum diversity of each antenna, considering the age, sex, and infection status of the insect. ^0^ *D* is equivalent to sensilla richness and is insensitive to abundance; ^1^ *D* is equivalent to the exponential of Shannon diversity and accounts for the effective number of abundant sensilla in the antenna; ^2^ *D* is equivalent to the inverse Simpson index and represents the effective number of dominant sensilla in each antenna. Additionally, we calculated Pielou's evenness index to assess the uniformity in the distribution of the different types of sensilla among the antennae.

Generalized linear models were constructed to evaluate whether there were significant differences in the diversity of sensilla per antenna and the abundance of sensilla per type and per antennal segment. In these models, infection status and its interaction with sex and age were considered as predictive factors. Furthermore, we employed generalized linear models to compare sensillum abundance per type and antennal segment among infected males and females and between uninfected males and females. We used a Gaussian error distribution for continuous response variables, such as ^1^ *D* and ^2^ *D* diversity and Pielou's evenness (J). A negative binomial error distribution was used for count‐type response variables such as the richness and abundance of sensilla. A negative binomial distribution was used instead of a Poisson distribution to correct for variance overdispersion in the count data (Zuur et al. 2009).

All analyses were performed in the R v.4.1.1 statistical environment (R Core Team 2021). We used the Vegan package (Oksanen et al. 2018) to calculate the diversity metrics and Hellinger distances, as well as to perform PERMANOVA and PERMADISP tests. Gaussian and negative binomial models were created using the stats (R Core Team 2021) and MASS (Venables and Ripley 2002) packages, respectively. The assumptions of normality and homoscedasticity of variance for models with a Gaussian distribution were evaluated using the performance R‐package (Lüdecke et al. 2021). The contrast between groups was conducted in each model with the package emmeans (Lenth 2023) at a 90% confidence level, correcting the *p* value for multiple tests with the false discovery rate method (fdr). Because the parasite can affect the bugs in different ways, depending on the species, developmental stage, and sex (see May‐Concha et al. 2022), the data may show significant variability. Therefore, a 90% confidence level may be more appropriate in exploratory studies like ours to detect subtle differences or patterns that are less evident with a 95% threshold, especially in contexts of high biological variability. Additionally, the FDR correction method provides an effective balance between identifying significant findings and minimizing Type I errors, that is, false positives (Waite and Campbell 2006).

## 3 Results

### 3.1 Generalities of Antennal Phenotype

**FIGURE 2:**
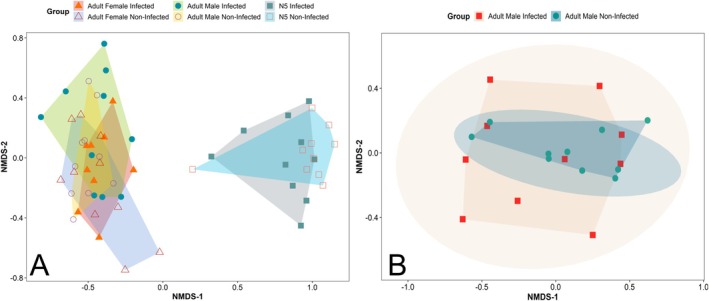
Non‐metric multidimensional scaling (NMDS) ordination depicting the composition of antennal phenotypes in *Triatoma pallidipenis*, based on Hellinger distances. Polygons determine groups based on developmental stage (N5 nymphs, adult males, and adult females) and infection condition (infected and uninfected); B Visualization of PERMADISP (permutational analysis of multivariate dispersions) results illustrating the spatial dispersion of multivariate data points for both infected and noninfected adult males, positioned beyond their respective ordination polygons in the NMDS plot. Complete test results are shown in Table S3.

Nymphs showed one mechanoreceptor (i.e., Bristles) type on the antennal pedicel, with an average of 126 sensilla per insect (± 12.60 SD; Table 2; Figure 2A and Table S1). On Flagellum 1 and Flagellum 2, basiconic sensilla were the least common, averaging 1.4 per insect (± 2.16 SD), and the flagellum with the highest abundance of sensilla was on Flagellum 2 (71 ± 59.87 SD) compared with Flagellum 1 (12 ± 14.95 SD) (Table S2).

**TABLE 2:** Mean abundance of sensilla by type, segment, sex, age, and infection status.

	Female infected	Female noninfected	Male infected	Male noninfected	N5 nymph infected	N5 nymph noninfected
P‐BR	187.11 (24.51)	154.00 (41.54)	184.80 (16.50)	170.50 (20.69)	119.70 (8.06)	132.90 (13.17)
P‐BA	1.78 (16.00)	3.00 (30.00)	4.00 (40.00)	7.70 (77.00)	0.00	0.00
P‐TK	73.22 (67.65)	63.10 (79.53)	138.80 (124.47)	98.00 (91.29)	0.00	0.00
P‐TH	86.00 (55.48)	79.10 (71.33)	88.70 (82.77)	123.10 (86.51)	0.00	0.00
FI‐BR	38.78 (8.57)	34.00 (6.75)	30.40 (9.26)	31.00 (5.01)	33.60 (2.59)	35.20 (3.61)
FI‐BA	30.00 (18.30)	22.30 (12.30)	36.30 (19.38)	25.40 (14.68)	1.90 (2.42)	0.90 (1.85)
FI‐TK	180.33 (64.21)	182.10 (88.42)	213.20 (96.00)	210.70 (89.85)	7.40 (13.65)	4.70 (11.77)
FI‐TH	247.67 (99.12)	229.10 (99.34)	169.00 (94.33)	196.70 (75.37)	7.40 (12.05)	7.20 (22.07)
FII‐BR	23.78 (14.07)	26.90 (32.44)	24.50 (10.11)	19.10 (8.06)	20.60 (10.52)	18.30 (7.18)
FII‐BA	33.22 (24.87)	22.40 (9.26)	32.00 (8.84)	29.70 (14.24)	14.10 (12.26)	22.70 (11.07)
FII‐TK	131.56 (85.20)	94.30 (50.41)	155.10 (69.97)	148.30 (65.20)	127.50 (67.99)	117.50 (54.78)
FII‐TH	178.67 (60.19)	184.40 (71.66)	135.90 (76.35)	159.00 (59.95)	125.20 (55.24)	120.30 (71.43)

*Note:* The standard deviation (SD) is indicated in parentheses to the right of each average value. Pedicel (P), flagellum 1 (F I), flagellum 2 (F II); bristles (BR), basiconic sensilla (BA), thick‐walled trichoid (TK), thin‐walled trichoid (TH). Complete count data are given in Tables S1 and S2.

While adult males had an average of 1216 sensilla (± 233.58 SD) per antenna, adult females had an average of 1150 sensilla (± 250.68 SD) (Figure 2B). Overall, the highest abundance of sensilla in adults was on Flagellum 1 (females: 481 ± 105.69 SD; males: 456 ± 96.93 SD). The most abundant sensilla in both sexes was the thin‐walled trichoid of Flagellum 1, with an average of 210 sensilla per antenna (± 93.70 SD). In contrast, the basiconic sensilla on the Pedicel segment were the least frequent, averaging 4.18 per antenna (± 5.73 SD).

### 3.2 Antennal Sensilla in Infected and Noninfected Bugs

#### 3.2.1 Multivariate Analysis

**FIGURE 3:**
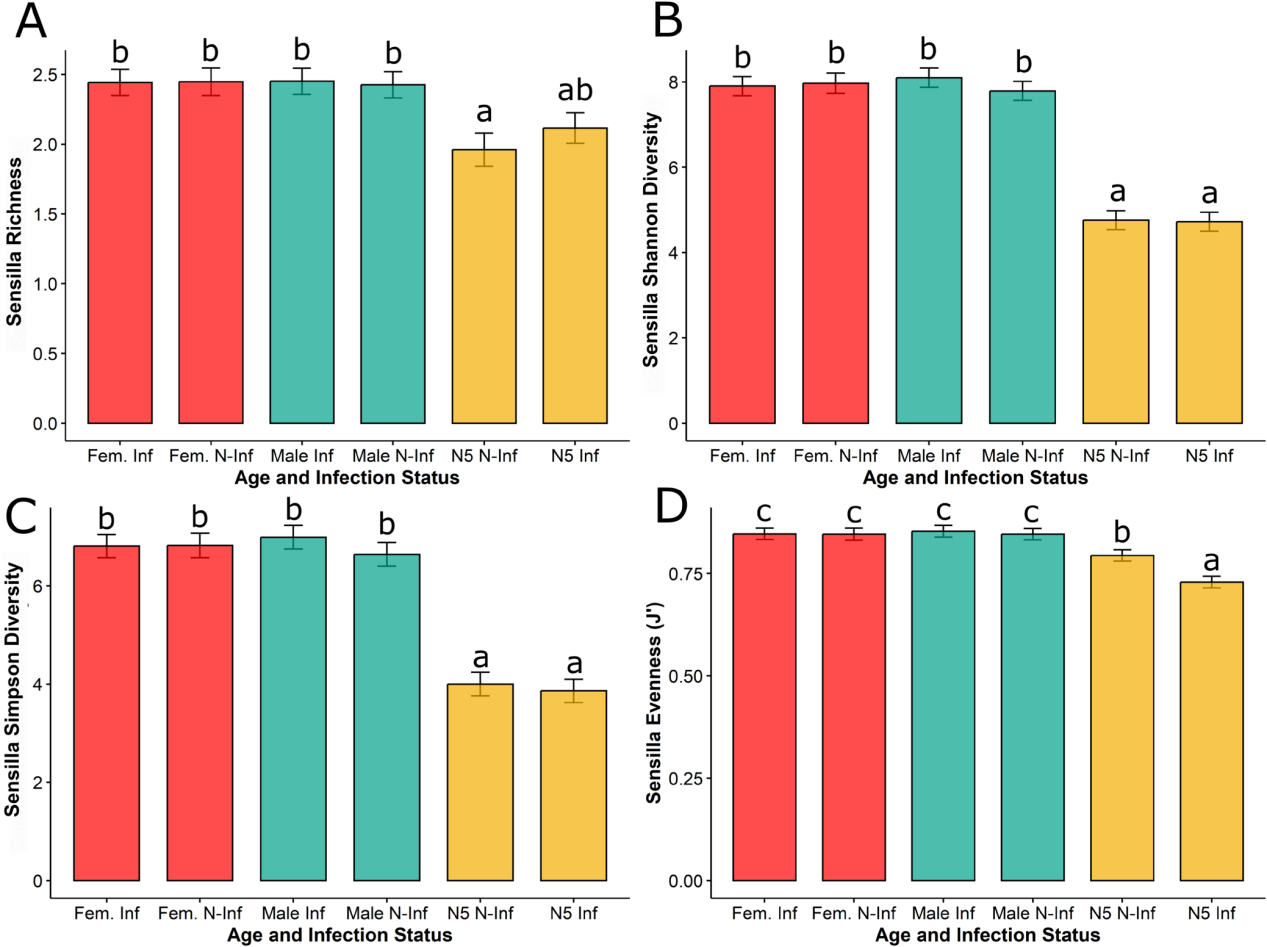
Bar plots of richness and diversity of antennal sensilla in *Triatoma pallidipennis* , categorized by developmental stage (N5–Nymph, adult female, and male) and infection status (infected, noninfected) (A) Sensilla richness; (B) Sensilla Shannon diversity; (C) Sensilla Simpson diversity, sensilla's Pileou evenness (J'). Error bars represent the standard deviation. Statistically significant differences are indicated by different letters above the bars (*p* < 0.1). Complete test results are shown in Table S4.

The PERMANOVA test indicated that the composition of the antennal phenotype was affected only by age (*p* value < 0.01), while infection status (*p* value = 0.71) and the interaction between infection status and age (*p* value = 0.70; Figure 3A and Table S3a–d) did not have a significant association with the antennal phenotype. The PERMADISP test showed results approaching statistical significance in the multivariate dispersion of the antennal phenotype of infected and noninfected males (*p* value = 0.06; Figure 3B) but not between infected and noninfected females or nymphs (Figure 3B).

#### 3.2.2 Diversity and Evenness of Sensillum Phenotype

Age was the sole factor that affected the richness (^0^ *D*) and diversity (^1^ *D* and ^2^ *D*) of each sensillum type (Table S4a–d). Pielou's J' analysis showed that age, infection status, and their interaction influenced the evenness of antennal sensilla.

#### 3.2.3 Univariate Analyses

The abundance of the four sensillum types between the antennal segments of nymphs and females was significantly different. However, no significant differences were observed in the thin‐walled trichoids of males (Table 3 and Table S5a–c). Infection status only affected the number of bristles in nymphs and males (Table 3 and Table S6). The interaction of the antennal segment between noninfected males and females had a significant association with the abundance of the four sensillum types analyzed (Table 3 and Table S6). However, for infected males and females, the only significant difference was in the number of sensilla per segment (Table 3 and Table S6).

**TABLE 3:** Univariate model results comparing the abundance of sensilla types (BA, basiconic; BR, bristle; TH, thin‐walled trichoid; TK, thick‐walled trichoid) per antennal segment (AS) between nymphs, females, and males based on their infection status (IC, infected INF; not Infected N‐INF).

Insect group	BR	BA	TK	TH
N5 nymph (AS)	< 0.01	< 0.01	< 0.01	< 0.01
N5 nymph (IC)	0.05	—	—	—
N5 nymph (AS * IC)	—	—	—	—
Adult female (AS)	< 0.01	< 0.01	< 0.01	< 0.01
Adult female (IC)	—	—	—	—
Adult female (AS * IC)	—	—	—	—
Adult male (AS)	< 0.01	< 0.01	0.05	—
Adult male (IC)	0.08	—	—	—
Adult male (AS * IC)	—	—	—	—
Male vs. female (N‐INF AS)	< 0.01	< 0.01	< 0.01	< 0.01
Male vs. female (N‐INF)	—	< 0.01	—	—
Male vs. female (N‐INF AS * Sex)	—	< 0.01	—	—
Male vs. female (INF AS)	< 0.01	< 0.01	0.06	< 0.01
Male vs. female (INF)	—	—	—	—
Male vs. female (INF AS * Sex)	—	—	—	—

*Note:* Complete model results are shown in the Supporting Information. Complete univariate model results are shown in the Supporting Information. (Tables S3a–d, S5 and S6).

## 4 Discussion

Our study highlights the effects of *Trypanosoma cruzi* infection on the antennal phenotype of *T. pallidipennis* , a key vector of *T. cruzi* in Mexico (Meraz‐Medina et al. 2024), spanning both their 5th nymphal and adult stages. To our knowledge, this research is the first to document experimentally the infection effects in laboratory‐reared juveniles and to elucidate the parasite's influence on sexual dimorphism in adult specimens. We identified a significant variation of sensillum abundance and distribution across the antennal segments among adult males, females, and nymphs. The proximal flagellum exhibited the highest sensillum abundance, followed by the distal flagellum, with the pedicel showing the lowest abundance. However, the distribution of thin‐walled trichoids remained consistently high across all antennal segments in both infected and uninfected males. This is similar to what May‐Concha et al. (2016) described in *Triatoma dimidiata* . The function of these trichoids is unclear, yet a high density of thin‐walled trichoids in males has been associated with an enhanced detection of sex pheromones and the ability to survive in less stable habitats (May‐Concha et al. 2016; Montes de Oca‐Aguilar et al. 2022). It has also been suggested that such trichoids improve males' efficiency in locating mates as well as hosts (De La Carbajal Fuente and Catalá 2002; Catalá et al. 2005). Whether these functions operate in *T. pallidipennis* is unclear.

Univariate and multivariate analyses showed differences between nymphs and adults. Nymphs were characterized by a reduced abundance of sensilla, as well as a complete absence of chemoreceptors on the pedicel. Biologically, the greater abundance of sensilla on the antennae of adults likely reflects unique adaptations used during reproduction or enhanced activities such as dispersal and/or foraging (Abrahan et al. 2011; da Rosa et al. 1999; Galvez‐Marroquin et al. 2018; May‐Concha et al. 2016). Nevertheless, the absence of chemoreceptors on the pedicel may indicate that the juvenile stage of this species is adapted to stable but limited habitats (Catala 1997), as observed in *Triatoma sordida* or *Triatoma pseudomaculata* (De La Carbajal Fuente and Catalá 2002).

Infected males showed a less homogeneous dispersion of the sensillum types than noninfected males. Specifically, infected males had a proportionally higher presence of thick‐walled trichoids in comparison with thin‐walled trichoids. Unlike the thin‐walled trichoids, the chemoreceptive function of thick‐walled trichoids remains unclear (Guerenstein 1999). Bernard (1974) suggested that thick‐walled trichoids might be linked to pheromone perception, which plays a role in both sexual and aggregation behaviors in triatomines (Cruz‐López et al. 2001). Interestingly, there are differences in the qualitative composition of the aggregation pheromone in *T. pallidipennis* that depend on infection status: while n‐undecane, n‐dodecane, n‐tridecane, n‐tetradecane, dodecanol, benzothiazole, and 2‐(E)‐nonenal are produced by infected insects, nonanoic acid, caprolactam, 2‐methylquinazoline, 2,4‐dimethylquinazoline, and dodecanoic acid were found in noninfected insects (Alavez‐Rosas et al. 2024). If parasite manipulation influences pheromone production and detection, infected individuals could be expected to respond more effectively to pheromone‐emitting counterparts (Córdoba‐Aguilar 2020). These findings highlight the need for future studies to explore whether thick‐walled trichoids are indeed involved in pheromone detection.

Infection status did not significantly influence the richness and diversity of antennal sensilla, in contrast to the effect of age. It is thought that differences might be due to the age of the insect (i.e., adults vs. nymphs), as the increase in abundance and diversity of sensilla is influenced by the sensorial requirements of insects in their adult stage (Catalá and Schofield 1994; May‐Concha et al. 2015). Conversely, according to the Pielou evenness index results, the distribution of various types of antennal sensilla in infected nymphs was less uniform compared to noninfected nymphs.

We found a lower abundance of chemosensilla in females, especially basiconic sensilla, which were significantly less prevalent in noninfected females than in noninfected males. Similar findings have been reported by May‐Concha et al. (2016). Surprisingly, the differences in the antennal phenotype between sexes fade in infected individuals. This change seems to result from an increase in chemosensilla, especially thin‐walled trichoids in females, leading to a similar abundance to that of males. According to Arroyo et al. (2007) and Catalá et al. (2005), thin‐walled trichoids are associated with the ability of insects to disperse and survive in diverse habitat conditions. Thus, our findings suggest that *T. cruzi* may have the potential to homogenize the antennae of adult kissing bugs, resulting in optimized antennal phenotypes for mobility, reproduction, and host detection.

Studies on other insects have shown that parasites can alter the host's central nervous system, resulting in changes in the immune response, behavior, and host development (Herbison 2017; Malik et al. 2019; Melo et al. 2006). MicroRNAs, which are known to be significantly modulated by blood‐feeding in vector arthropods (Cocchiaro et al. 2008; Melo et al. 2006), may be involved in this association between the parasite and the insect system. In Chagas disease vectors, the relationship between *T. cruzi* and the Triatominae is so close that decapitation disrupts the development of *T. cruzi* infection as the lack of neural signals disrupts the structure of the insect's intestinal epithelium, hindering the parasite's ability to develop (Gonzalez et al. 1999). Other effects are less direct, as parasites may affect sensillum phenotypes by imposing physiological stress on hosts via the nutrients the former consume (Lawrence 1986; Marcogliese 2004). Another explanation is the energy allocated to the immune response by infected hosts that may be traded off with the energy required for sensory functions (Pimentel Melo et al. 2020; Samaddar et al. 2020). Finally, our results suggest that the infection status may favor the emergence of sensilla associated with the adult stage, as indicated by our results, where the richness of sensilla in infected nymphs resembled that of adult individuals, potentially causing less homogeneity in their distribution. The metabolic pressure and physiological stress incurred by the infection effect on the nymphs could be the reason for this increase.

It is important to acknowledge some of the limitations of our study. First, multiple lines of evidence suggest that the effects of infection on triatomine hosts can vary significantly depending on factors such as species, age, sex, and environmental conditions (Guarneri and Schaub 2021). In particular, stressful environments may trigger adverse physiological or behavioral responses, while in other cases, infections may have no observable impact on triatomine populations (Schaub 2021). Furthermore, because our study was conducted on laboratory‐reared populations under controlled conditions, the ecological relevance of the parasite's effects on antennal phenotype may be limited, as these effects could manifest differently in natural environments, as suggested by May‐Concha et al. (2022). Second, our study did not directly investigate the functional roles of these antennal phenotypes in the ecology and behavior of *T. pallidipennis* . However, recent evidence indicates that antennal phenotypes are linked to various sensory capacities across multiple insect taxa (Elgar et al. 2018; Guo et al. 2022; McKenzie et al. 2021; Polidori et al. 2020). For instance, May‐Concha et al. (2018) showed that the olfactory sensitivity of *Triatoma dimidiata* to different odors depends on the abundance of chemosensilla present on their antenna. Therefore, the variation in antennal phenotypes observed in *T. pallidipennis* could potentially influence their ecological interactions and behavioral responses, particularly in host‐seeking and environmental sensing.

## Author Contributions

**Jose D. Rivera‐Duarte:** conceptualization (equal), data curation (equal), formal analysis (equal), visualization (equal), writing – original draft (equal), writing – review and editing (equal). **Irving Jesús May‐Concha:** data curation (equal), investigation (equal), validation (equal), visualization (equal). **Reyna Vargas‐Abasolo:** data curation (equal), formal analysis (equal), visualization (equal). **Mayab X. Martínez‐Castaneira:** data curation (equal), investigation (equal), methodology (equal). **Manuel Edday Farfán‐Beltrán:** investigation (equal), methodology (equal). **Berenit Mendoza‐Garfias:** investigation (equal), methodology (equal). **Any Laura Flores‐Villegas:** investigation (equal), methodology (equal). **Alex Córdoba‐Aguilar:** conceptualization (equal), funding acquisition (equal), investigation (equal), methodology (equal), project administration (equal), resources (equal), writing – original draft (equal), writing – review and editing (equal).

## Conflicts of Interest

The authors declare no conflicts of interest.

## Supporting information


Tables S1–S6.

## Data Availability

The supporting data that accompany this paper can be accessed for free from: https://doi.org/10.6084/m9.figshare.28465670.v1.
