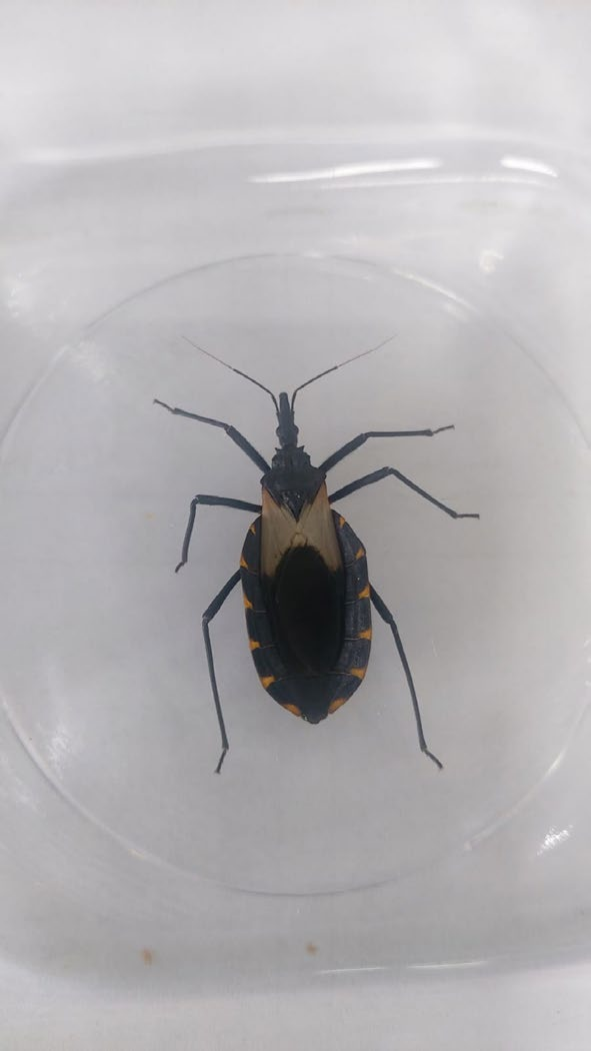# Correction to **“**Rewiring the Vehicle: *Trypanosoma cruzi* Parasites Alter the Antennae of Their Triatomine Hosts”

**DOI:** 10.1002/ece3.71220

**Published:** 2025-04-01

Rivera‐Duarte, J.D., May‐Concha, I.J., Vargas‐Abasolo, R., Martínez‐Castaneira, M.X., Farfán‐Beltrán, M.E., Mendoza‐Garfias, B., Flores‐Villegas, A.L., and Córdoba‐Aguilar, A. (2025), Rewiring the Vehicle: *Trypanosoma cruzi* Parasites Alter the Antennae of Their Triatomine Hosts. *Ecology and Evolution*, 15: e71164. https://doi.org/10.1002/ece3.71164.

Please note that figure 1 corresponds to a different insect species. The correct species ( *Triatoma pallidipennis* ) is shown below.

We apologize for this error.