# Supplementary material for: Rewiring the Vehicle: Trypanosoma cruzi Parasites Alter the Antennae of Their Triatomine Hosts
Source: Ecol Evol. 2025 Mar 23;15(3):e71164. doi: 10.1002/ece3.71164 (PMC11930763; doi:10.1002/ece3.71164)
Supplement: Supplementary file 1 — Tables S1–S6. [file ECE3-15-e71164-s001.docx]

## Supplementary material

**Table S1**. Antennal sensila abundance by antennal segment (P: pedicel; FI: Flagellum I, FII: Flagellum II) development stage (N5-Nymph, Adult female, andult male) and infection condition (Infected and non-infected). Sensilla types: BR Bristles, BA Basiconic, TK Thick-walled trichoid; TH Thin-walled trichoid.

| **Insect group** | **P-BR** | **P-BA** | **P-TK** | **P-TH** | **FI-BR** | **FI-BA** | **FI-TK** | **FI-TH** | **FII-BR** | **FII-BA** | **FII-TK** | **FII-TH** |
| --- | --- | --- | --- | --- | --- | --- | --- | --- | --- | --- | --- | --- |
| **Female Infected** | 197 | 7 | 132 | 89 | 44 | 63 | 227 | 240 | 27 | 40 | 152 | 164 |
| **Female Infected** | 194 | 4 | 28 | 157 | 40 | 16 | 146 | 195 | 20 | 20 | 99 | 131 |
| **Female Infected** | 152 | 2 | 82 | 48 | 35 | 17 | 204 | 148 | 12 | 25 | 194 | 108 |
| **Female Infected** | 205 | 0 | 2 | 47 | 33 | 3 | 91 | 198 | 20 | 5 | 7 | 124 |
| **Female Infected** | 217 | 1 | 90 | 142 | 36 | 39 | 310 | 227 | 9 | 81 | 298 | 128 |
| **Female Infected** | 142 | 1 | 72 | 105 | 35 | 19 | 161 | 208 | 13 | 15 | 76 | 215 |
| **Female Infected** | 193 | 1 | 26 | 40 | 25 | 36 | 118 | 231 | 50 | 10 | 143 | 243 |
| **Female Infected** | 185 | 0 | 13 | 146 | 54 | 46 | 184 | 491 | 20 | 56 | 57 | 270 |
| **Female Infected** | 199 | 0 | 214 | 0 | 47 | 31 | 182 | 291 | 43 | 47 | 158 | 225 |
| **Female Non-Infected** | 147 | 4 | 6 | 146 | 36 | 9 | 83 | 214 | 7 | 13 | 50 | 190 |
| **Female Non-Infected** | 170 | 0 | 0 | 18 | 46 | 23 | 10 | 240 | 25 | 37 | 20 | 182 |
| **Female Non-Infected** | 214 | 10 | 72 | 32 | 24 | 25 | 313 | 214 | 37 | 30 | 176 | 146 |
| **Female Non-Infected** | 160 | 7 | 56 | 204 | 30 | 12 | 153 | 204 | 12 | 23 | 65 | 99 |
| **Female Non-Infected** | 51 | 0 | 0 | 0 | 40 | 42 | 197 | 488 | 116 | 12 | 180 | 358 |
| **Female Non-Infected** | 177 | 0 | 196 | 12 | 28 | 31 | 225 | 213 | 17 | 20 | 83 | 196 |
| **Female Non-Infected** | 151 | 5 | 38 | 110 | 35 | 13 | 206 | 225 | 12 | 13 | 84 | 104 |
| **Female Non-Infected** | 166 | 2 | 48 | 106 | 33 | 20 | 239 | 131 | 17 | 34 | 108 | 184 |
| **Female Non-Infected** | 140 | 0 | 0 | 142 | 40 | 9 | 140 | 126 | 14 | 15 | 92 | 176 |
| **Female Non-Infected** | 164 | 2 | 215 | 21 | 28 | 39 | 255 | 236 | 12 | 27 | 85 | 209 |
| **Male Infected** | 197 | 20 | 368 | 137 | 37 | 56 | 258 | 265 | 27 | 28 | 205 | 54 |
| **Male Infected** | 173 | 0 | 15 | 157 | 25 | 2 | 115 | 104 | 14 | 18 | 59 | 106 |
| **Male Infected** | 149 | 3 | 3 | 140 | 35 | 18 | 173 | 204 | 18 | 19 | 86 | 213 |
| **Male Infected** | 198 | 7 | 310 | 2 | 34 | 56 | 192 | 234 | 34 | 40 | 44 | 228 |
| **Male Infected** | 181 | 4 | 170 | 0 | 38 | 43 | 229 | 232 | 21 | 37 | 223 | 116 |
| **Male Infected** | 194 | 0 | 160 | 0 | 33 | 19 | 364 | 82 | 17 | 44 | 186 | 82 |
| **Male Infected** | 181 | 1 | 84 | 128 | 44 | 29 | 206 | 186 | 16 | 32 | 172 | 124 |
| **Male Infected** | 209 | 0 | 0 | 66 | 16 | 49 | 171 | 296 | 42 | 27 | 152 | 151 |
| **Male Infected** | 182 | 0 | 147 | 237 | 17 | 59 | 63 | 73 | 38 | 39 | 174 | 258 |
| **Male Infected** | 184 | 5 | 131 | 20 | 25 | 32 | 361 | 14 | 18 | 36 | 250 | 27 |
| **Male Non-Infected** | 191 | 0 | 139 | 107 | 29 | 29 | 311 | 184 | 17 | 32 | 190 | 167 |
| **Male Non-Infected** | 118 | 4 | 15 | 231 | 31 | 34 | 142 | 140 | 10 | 20 | 107 | 124 |
| **Male Non-Infected** | 162 | 0 | 14 | 151 | 29 | 8 | 134 | 89 | 15 | 4 | 62 | 96 |
| **Male Non-Infected** | 178 | 17 | 51 | 229 | 34 | 26 | 298 | 200 | 21 | 38 | 226 | 141 |
| **Male Non-Infected** | 177 | 18 | 55 | 226 | 25 | 15 | 72 | 374 | 35 | 15 | 58 | 316 |
| **Male Non-Infected** | 189 | 0 | 139 | 99 | 23 | 34 | 279 | 189 | 14 | 41 | 151 | 172 |
| **Male Non-Infected** | 180 | 7 | 76 | 109 | 33 | 9 | 210 | 206 | 14 | 26 | 192 | 165 |
| **Male Non-Infected** | 173 | 9 | 278 | 0 | 32 | 34 | 333 | 165 | 24 | 43 | 200 | 147 |
| **Male Non-Infected** | 174 | 20 | 208 | 1 | 41 | 54 | 188 | 250 | 29 | 51 | 214 | 121 |
| **Male Non-Infected** | 163 | 2 | 5 | 78 | 33 | 11 | 140 | 170 | 12 | 27 | 83 | 141 |
| **N5 Infected** | 123 | 0 | 0 | 0 | 35 | 0 | 1 | 1 | 16 | 6 | 52 | 202 |
| **N5 Infected** | 112 | 0 | 0 | 0 | 30 | 5 | 15 | 19 | 29 | 4 | 162 | 50 |
| **N5 Infected** | 129 | 0 | 0 | 0 | 32 | 1 | 7 | 3 | 37 | 0 | 188 | 83 |
| **N5 Infected** | 114 | 0 | 0 | 0 | 31 | 0 | 2 | 4 | 8 | 9 | 91 | 115 |
| **N5 Infected** | 112 | 0 | 0 | 0 | 33 | 1 | 2 | 2 | 14 | 24 | 78 | 196 |
| **N5 Infected** | 124 | 0 | 0 | 0 | 32 | 7 | 44 | 38 | 34 | 5 | 118 | 114 |
| **N5 Infected** | 111 | 0 | 0 | 0 | 37 | 3 | 0 | 4 | 16 | 10 | 20 | 181 |
| **N5 Infected** | 128 | 0 | 0 | 0 | 35 | 0 | 2 | 1 | 7 | 19 | 240 | 54 |
| **N5 Infected** | 113 | 0 | 0 | 0 | 33 | 0 | 0 | 1 | 27 | 39 | 148 | 147 |
| **N5 Infected** | 131 | 0 | 0 | 0 | 38 | 2 | 1 | 1 | 18 | 25 | 178 | 110 |
| **N5 Non-Infected** | 126 | 0 | 0 | 0 | 32 | 6 | 38 | 70 | 12 | 16 | 69 | 83 |
| **N5 Non-Infected** | 151 | 0 | 0 | 0 | 37 | 0 | 0 | 2 | 29 | 23 | 211 | 137 |
| **N5 Non-Infected** | 134 | 0 | 0 | 0 | 37 | 0 | 0 | 0 | 6 | 6 | 44 | 44 |
| **N5 Non-Infected** | 115 | 0 | 0 | 0 | 36 | 1 | 3 | 0 | 28 | 20 | 129 | 111 |
| **N5 Non-Infected** | 146 | 0 | 0 | 0 | 34 | 0 | 3 | 0 | 16 | 43 | 99 | 153 |
| **N5 Non-Infected** | 144 | 0 | 0 | 0 | 42 | 0 | 1 | 0 | 23 | 35 | 175 | 178 |
| **N5 Non-Infected** | 145 | 0 | 0 | 0 | 34 | 1 | 2 | 0 | 15 | 22 | 176 | 41 |
| **N5 Non-Infected** | 120 | 0 | 0 | 0 | 33 | 1 | 0 | 0 | 17 | 16 | 103 | 179 |
| **N5 Non-Infected** | 117 | 0 | 0 | 0 | 29 | 0 | 0 | 0 | 15 | 14 | 63 | 30 |
| **N5 Non-Infected** | 131 | 0 | 0 | 0 | 38 | 0 | 0 | 0 | 22 | 32 | 106 | 247 |

**Table S2**. Antennal sensilla diversity results per antenna of each insect group. Antennal sensilla richness (^0^*D*), Shannon diversity (^1^*D*), Simpson diversity (^2^*D*), Pielou Evenness (J')

| **Insect group** | **0D** | **1D** | **2D** | **J** |
| --- | --- | --- | --- | --- |
| **Female Infected** | 12 | 2.23 | 9.32 | 8.25 |
| **Female Infected** | 12 | 2.12 | 8.35 | 7.25 |
| **Female Infected** | 12 | 2.12 | 8.31 | 7.16 |
| **Female Infected** | 11 | 1.80 | 6.04 | 4.97 |
| **Female Infected** | 12 | 2.13 | 8.45 | 7.38 |
| **Female Infected** | 12 | 2.10 | 8.19 | 7.07 |
| **Female Infected** | 12 | 2.05 | 7.79 | 6.53 |
| **Female Infected** | 11 | 1.97 | 7.20 | 5.58 |
| **Female Infected** | 10 | 2.09 | 8.06 | 7.22 |
| **Female Non-Infected** | 12 | 1.97 | 7.15 | 6.03 |
| **Female Non-Infected** | 10 | 2.00 | 7.37 | 6.38 |
| **Female Non-Infected** | 12 | 2.09 | 8.05 | 6.64 |
| **Female Non-Infected** | 12 | 2.10 | 8.19 | 6.95 |
| **Female Non-Infected** | 12 | 2.18 | 8.84 | 7.91 |
| **Female Non-Infected** | 11 | 2.04 | 7.68 | 6.72 |
| **Female Non-Infected** | 12 | 2.07 | 7.93 | 6.66 |
| **Female Non-Infected** | 12 | 2.13 | 8.44 | 7.23 |
| **Female Non-Infected** | 10 | 2.02 | 7.55 | 6.84 |
| **Female Non-Infected** | 12 | 2.05 | 7.80 | 6.73 |
| **Male Infected** | 12 | 2.14 | 8.49 | 7.16 |
| **Male Infected** | 11 | 2.02 | 7.57 | 6.56 |
| **Male Infected** | 12 | 2.04 | 7.67 | 6.69 |
| **Male Infected** | 12 | 2.05 | 7.76 | 6.60 |
| **Male Infected** | 11 | 2.08 | 7.99 | 7.09 |
| **Male Infected** | 10 | 1.94 | 6.97 | 5.64 |
| **Male Infected** | 12 | 2.18 | 8.87 | 7.94 |
| **Male Infected** | 10 | 2.02 | 7.52 | 6.44 |
| **Male Infected** | 11 | 2.15 | 8.56 | 7.40 |
| **Male Infected** | 12 | 1.86 | 6.45 | 4.90 |
| **Male Non-Infected** | 11 | 2.13 | 8.39 | 7.36 |
| **Male Non-Infected** | 12 | 2.10 | 8.16 | 6.97 |
| **Male Non-Infected** | 11 | 2.02 | 7.53 | 6.53 |
| **Male Non-Infected** | 12 | 2.14 | 8.53 | 7.33 |
| **Male Non-Infected** | 12 | 1.98 | 7.27 | 5.71 |
| **Male Non-Infected** | 11 | 2.14 | 8.52 | 7.55 |
| **Male Non-Infected** | 12 | 2.13 | 8.38 | 7.42 |
| **Male Non-Infected** | 11 | 2.05 | 7.73 | 6.64 |
| **Male Non-Infected** | 12 | 2.16 | 8.63 | 7.60 |
| **Male Non-Infected** | 12 | 2.06 | 7.83 | 6.80 |
| **N5 Infected** | 8 | 1.38 | 3.97 | 3.16 |
| **N5 Infected** | 9 | 1.69 | 5.43 | 4.16 |
| **N5 Infected** | 8 | 1.51 | 4.52 | 3.76 |
| **N5 Infected** | 8 | 1.52 | 4.59 | 3.93 |
| **N5 Infected** | 9 | 1.52 | 4.55 | 3.62 |
| **N5 Infected** | 9 | 1.87 | 6.49 | 5.56 |
| **N5 Infected** | 8 | 1.41 | 4.09 | 3.09 |
| **N5 Infected** | 8 | 1.36 | 3.88 | 3.01 |
| **N5 Infected** | 7 | 1.60 | 4.93 | 4.33 |
| **N5 Infected** | 9 | 1.56 | 4.76 | 4.01 |
| **N5 Non-Infected** | 9 | 1.91 | 6.76 | 5.78 |
| **N5 Non-Infected** | 7 | 1.52 | 4.59 | 3.92 |
| **N5 Non-Infected** | 6 | 1.38 | 3.97 | 3.16 |
| **N5 Non-Infected** | 8 | 1.62 | 5.06 | 4.39 |
| **N5 Non-Infected** | 7 | 1.58 | 4.88 | 4.22 |
| **N5 Non-Infected** | 7 | 1.55 | 4.72 | 4.13 |
| **N5 Non-Infected** | 8 | 1.46 | 4.30 | 3.42 |
| **N5 Non-Infected** | 7 | 1.48 | 4.41 | 3.75 |
| **N5 Non-Infected** | 6 | 1.50 | 4.50 | 3.62 |
| **N5 Non-Infected** | 6 | 1.48 | 4.38 | 3.59 |

**Table S3a**. PERMANOVA test result. All groups

|  | DF | Sum of Squares | R^2^ | F | p-value |
| --- | --- | --- | --- | --- | --- |
| Age | 2 | 5.95 | 0.60 | 41.54 | < 0.01 |
| Infection status | 1 | 0.03 | 0.00 | 0.45 | 0.71 |
| Age * Infection status | 2 | 0.09 | 0.01 | 0.63 | 0.70 |
| Residuals | 53 | 3.80 | 0.38 |  |  |

**Table S3b**. PERMANOVA test result. N5-nymphs

|  | DF | Sum of Squares | R^2^ | F | p-value |
| --- | --- | --- | --- | --- | --- |
| Infection status | 1 | >0.01 | >0.01 | 0.5 | 0.99 |
| Residual | 18 | 0.20 | 0.01 |  |  |

**Table S3c**. PERMANOVA test result. Adult females

|  | DF | Sum of Squares | R^2^ | F | p-value |
| --- | --- | --- | --- | --- | --- |
| Infection status | 1 | 0.01 | 0.01 | 1.20 | 0.28 |
| Residual | 17 | 0.18 | 0.01 |  |  |

**Table S3d**. PERMANOVA test result. Adult males

|  | DF | Sum of Squares | R^2^ | F | p-value |
| --- | --- | --- | --- | --- | --- |
| Infection status | 1 | 0.03 | 0.03 | 3.48 | 0.06 |
| Residual | 18 | 0.16 | 0.01 |  |  |

**Table S4a**. Sensilla richness (^0^*D*) test results

|  | DF | F.ratio | ChisSq | p-value |
| --- | --- | --- | --- | --- |
| Age | 2 | 9.27 | 18.53 | >0.01 |
| Infection status | 1 | 0.29 | 0.29 | 0.59 |
| Age * Infection status | 2 | 0.41 | 0.82 | 0.66 |

**Table S4b**. Sensilla Shannon Diversity (^1^*D*) test results

|  | DF | F.ratio | ChisSq | p-value |
| --- | --- | --- | --- | --- |
| Age | 2 | 53 | 134.35 | >0.01 |
| Infection status | 1 | 53 | 0.25 | 0.62 |
| Age * Infection status | 2 | 53 | 0.37 | 0.69 |

**Table S4c**. Sensilla Simpson Diversity (^2^*D*) test results

|  | DF | F.ratio | ChisSq | p-value |
| --- | --- | --- | --- | --- |
| Age | 2 | 53 | 97.04 | >0.01 |
| Infection status | 1 | 53 | 0.65 | 0.42 |
| Age * Infection status | 2 | 53 | 0.29 | 0.75 |

**Table S4d**. Sensilla Pileou Evenness (J*'*) test results

|  | DF | F.ratio | ChisSq | p-value |
| --- | --- | --- | --- | --- |
| Age | 2 | 53 | 25.10 | >0.01 |
| Infection status | 1 | 53 | 4.41 | 0.04 |
| Age * Infection status | 2 | 53 | 3.14 | 0.05 |

**Table S5a**. Univariate test results. N5-Nymphs. Bristles (Br), Basiconic (Ba), Thick-walled trichoids (TK), Thin-walled trichoids (Th).

| **Br N5-Nymph** | | **DF** | | **Deviance** | **Res DF** | | **Res. Dev** | | **p-value** | | |
| --- | --- | --- | --- | --- | --- | --- | --- | --- | --- | --- | --- |
| Antennal segment | | 2 | | 2011.25 | 57 | | 103.57 | | <0.01 | | |
| Infection status | | 1 | | 3.80 | 56 | | 99.76 | | 0.05 | | |
| Antennal segment * Infection status | | 2 | | 4.17 | 54 | | 95.60 | | 0.12 | | |
| **Ba N5-Nymph** | **DF** | | **Deviance** | | | **Res DF** | | **Res. Dev** | | **p-value** |  |
| Antennal segment | 2 | | 181.51 | | | 57 | | 51.47 | | <0.01 |  |
| Infection status | 1 | | 0.06 | | | 56 | | 51.41 | | 0.81 |  |
| Antennal segment * Infection status | 2 | | 3.61 | | | 54 | | 47.81 | | 0.16 |  |
| **TK N5-Nymph** | **DF** | | **Deviance** | | | **Res DF** | | **Res. Dev** | | **p-value** |  |
| Antennal segment | 2 | | 191.84 | | | 57 | | 48.63 | | <0.01 |  |
| Infection status | 1 | | 0.50 | | | 56 | | 48.13 | | 0.48 |  |
| Antennal segment * Infection status | 2 | | 0.27 | | | 54 | | 47.86 | | 0.88 |  |
| **TH N5-Nymph** | **DF** | | **Deviance** | | | **Res DF** | | **Res. Dev** | | **p-value** |  |
| Antennal segment | 2 | | 149.34 | | | 57 | | 47.25 | | <0.01 |  |
| Infection status | 1 | | 0.01 | | | 56 | | 47.24 | | 0.93 |  |
| Antennal segment * Infection status | 2 | | 0.00 | | | 54 | | 47.24 | | 0.99 |  |

**Table S5b**. Univariate test results. Adult females. Bristles (Br), Basiconic (Ba), Thick-walled trichoids (TK), Thin-walled trichoids (Th).

| **Br Female** | | **DF** | | **Deviance** | **Res DF** | | **Res. Dev** | | **p-value** | | |
| --- | --- | --- | --- | --- | --- | --- | --- | --- | --- | --- | --- |
| Antennal segment | | 2 | | 231.82 | 54 | | 62.25 | | <0.01 | | |
| Infection status | | 1 | | 0.46 | 53 | | 61.79 | | 0.50 | | |
| Antennal segment * Infection status | | 2 | | 1.41 | 51 | | 60.38 | | 0.49 | | |
| **Ba Female** | **DF** | | **Deviance** | | | **Res DF** | | **Res. Dev** | | **p-value** |  |
| Antennal segment | 2 | | 100.27 | | | 54 | | 73.54 | | <0.01 |  |
| Infection status | 1 | | 0.80 | | | 53 | | 72.74 | | 0.37 |  |
| Antennal segment * Infection status | 2 | | 3.49 | | | 51 | | 69.25 | | 0.17 |  |
| **TK Female** | **DF** | | **Deviance** | | | **Res DF** | | **Res. Dev** | | **p-value** |  |
| Antennal segment | 2 | | 10.73 | | | 54 | | 68.63 | | <0.01 |  |
| Infection status | 1 | | 0.42 | | | 53 | | 68.21 | | 0.52 |  |
| Antennal segment * Infection status | 2 | | 0.33 | | | 51 | | 67.88 | | 0.85 |  |
| **TH Female** | **DF** | | **Deviance** | | | **Res DF** | | **Res. Dev** | | **p-value** |  |
| Antennal segment | 2 | | 24.79 | | | 54 | | 68.06 | | <0.01 |  |
| Infection status | 1 | | 0.06 | | | 53 | | 68.00 | | 0.80 |  |
| Antennal segment * Infection status | 2 | | 0.09 | | | 51 | | 67.90 | | 0.95 |  |

**Table S5c**. Univariate test results. Adult Males. Bristles (Br), Basiconic (Ba), Thick-walled trichoids (TK), Thin-walled trichoids (Th).

| **Br Male** | | **DF** | | **Deviance** | **Res DF** | | **Res. Dev** | | **p-value** | | |
| --- | --- | --- | --- | --- | --- | --- | --- | --- | --- | --- | --- |
| Antennal segment | | 2 | | 1562.13 | 57 | | 83.20 | | <0.01 | | |
| Infection status | | 1 | | 3.02 | 56 | | 80.18 | | 0.08 | | |
| Antennal segment * Infection status | | 2 | | 3.14 | 54 | | 77.05 | | 0.21 | | |
| **Ba Male** | **DF** | | **Deviance** | | | **Res DF** | | **Res. Dev** | | **p-value** |  |
| Antennal segment | 2 | | 50.21 | | | 57 | | 81.15 | | <0.01 |  |
| Infection status | 1 | | 0.02 | | | 56 | | 81.13 | | 0.89 |  |
| Antennal segment * Infection status | 2 | | 4.31 | | | 54 | | 76.82 | | 0.11 |  |
| **TK Male** | **DF** | | **Deviance** | | | **Res DF** | | **Res. Dev** | | **p-value** |  |
| Antennal segment | 2 | | 5.94 | | | 57 | | 69.18 | | 0.05 |  |
| Infection status | 1 | | 0.46 | | | 56 | | 68.72 | | 0.49 |  |
| Antennal segment * Infection status | 2 | | 0.59 | | | 54 | | 68.13 | | 0.74 |  |
| **TH Male** | **DF** | | **Deviance** | | | **Res DF** | | **Res. Dev** | | **p-value** |  |
| Antennal segment | 2 | | 3.87 | | | 57 | | 72.80 | | 0.14 |  |
| Infection status | 1 | | 0.88 | | | 56 | | 71.92 | | 0.35 |  |
| Antennal segment * Infection status | 2 | | 0.13 | | | 54 | | 71.79 | | 0.94 |  |

**Table S6**. Univariate test results male vs female, infected and non-infected. Bristles (Br), Basiconic (Ba), Thick-walled trichoids (TK), Thin-walled trichoids (Th).

| **Br Male vs Female (Non-Infected)** | | **DF** | | **Deviance** | **Res DF** | | **Res. Dev** | | **p-value** | | |
| --- | --- | --- | --- | --- | --- | --- | --- | --- | --- | --- | --- |
| Antennal segment | | 2 | | 307.68 | 57 | | 66.81 | | <0.01 | | |
| Sex | | 1 | | 0.82 | 56 | | 65.99 | | 0.36 | | |
| Antennal segment * Sex | | 2 | | 3.12 | 54 | | 62.87 | | 0.21 | | |
| **Ba Male Vs Female (Non-Infected)** | **DF** | | **Deviance** | | | **Res DF** | | **Res. Dev** | | **p-value** |  |
| Antennal segment | 2 | | 242.44 | | | 57 | | 531.51 | | <0.01 |  |
| Sex | 1 | | 130.19 | | | 56 | | 401.32 | | <0.01 |  |
| Antennal segment * Sex | 2 | | 318.09 | | | 54 | | 83.23 | | <0.01 |  |
| **TK Male vs Female (Non-Infected)** | **DF** | | **Deviance** | | | **Res DF** | | **Res. Dev** | | **p-value** |  |
| Antennal segment | 2 | | 10.37 | | | 57 | | 74.05 | | <0.01 |  |
| Sex | 1 | | 2.33 | | | 56 | | 71.72 | | 0.13 |  |
| Antennal segment * Sex | 2 | | 0.39 | | | 54 | | 71.32 | | 0.82 |  |
| **TH Male Vs Female (Non-Infected)** | **DF** | | **Deviance** | | | **Res DF** | | **Res. Dev** | | **p-value** |  |
| Antennal segment | 2 | | 10.82 | | | 57 | | 73.52 | | <0.01 |  |
| Sex | 1 | | 0.06 | | | 56 | | 73.46 | | 0.81 |  |
| Antennal segment * Sex | 2 | | 2.27 | | | 54 | | 71.20 | | 0.32 |  |
| **Br Male vs Female (Infected)** | **DF** | | **Deviance** | | | **Res DF** | | **Res. Dev** | | **p-value** |  |
| Antennal segment | 2 | | 794.32 | | | 54 | | 71.95 | | <0.01 |  |
| Sex | 1 | | 1.07 | | | 53 | | 70.87 | | 0.30 |  |
| Antennal segment * Sex | 2 | | 2.78 | | | 51 | | 68.09 | | 0.25 |  |
| **Ba Male Vs Female (Infected)** | **DF** | | **Deviance** | | | **Res DF** | | **Res. Dev** | | **p-value** |  |
| Antennal segment | 2 | | 2 | | | 86.06 | | 57 | | 73.58 |  |
| Sex | 1 | | 1 | | | 1.38 | | 56 | | 72.20 |  |
| Antennal segment * Sex | 2 | | 2 | | | 2.44 | | 54 | | 69.76 |  |
| **TK Male vs Female (Infected)** | **DF** | | **Deviance** | | | **Res DF** | | **Res. Dev** | | **p-value** |  |
| Antennal segment | 2 | | 5.51 | | | 57 | | 68.48 | | 0.06 |  |
| Sex | 1 | | 2.27 | | | 56 | | 66.21 | | 0.13 |  |
| Antennal segment * Sex | 2 | | 1.11 | | | 54 | | 65.10 | | 0.57 |  |
| **TH Male Vs Female (Non-Infected)** | **DF** | | **Deviance** | | | **Res DF** | | **Res. Dev** | | **p-value** |  |
| Antennal segment | 2 | | 10.03 | | | 57 | | 70.01 | | <0.01 |  |
| Sex | 1 | | 0.91 | | | 56 | | 69.10 | | 0.34 |  |
| Antennal segment * Sex | 2 | | 0.63 | | | 54 | | 68.47 | | 0.73 |  |
